# CT-Based body composition and nutritional status as predictors of early-treatment chemotherapy-related infections and hematologic toxicity in pediatric cancer: a prospective study

**DOI:** 10.1007/s00431-026-06882-x

**Published:** 2026-03-25

**Authors:** Beatriz Pereira de Carvalho, Isabella Caroline Santana Aleixo, Nathalia Farache Tostes, Nilian Carla Souza, Danúbia da Cunha Antunes Saraiva, Renata Brum Martucci

**Affiliations:** 1 Post-Graduated Program in Medical Science, Medical Science Faculty, State University of Rio de Janeiro, Rio de Janeiro, RJ Brazil; 2 Nutrition and Dietetics Sector, Cancer Hospital Unit I, National Cancer Institute (INCA), Rio de Janeiro, RJ Brazil; 3 Post-Graduated Program in Nutrition, Food and Health, Nutrition Institute, Rio de Janeiro State University, Rio de Janeiro, RJ Brazil; 4 Nutrition and Dietetic Sector, Cancer Hospital Unit I, National Cancer Institute (INCA), Rio de Janeiro, RJ Brazil; 5 Department of Applied Nutrition, Nutrition Institute, Rio de Janeiro State University, Rio de Janeiro, Brazil, R. São Francisco Xavier, 524 - Maracanã, Rio de Janeiro, RJ CEP21550-170 Brazil

**Keywords:** Pediatrics, Computed tomography, Cancer, Body composition, Chemotherapy, Toxicity

## Abstract

Chemotherapy-related toxicity has been associated with body composition, yet there is no consensus on which CT-derived muscle parameter or handgrip strength (HGS) best predicts toxicity in pediatric cancer patients. This study evaluated whether CT-derived body composition parameters and nutritional status independently predict early chemotherapy-related infections and hematologic toxicity in children and adolescents with cancer. In this prospective observational cohort, patients aged 7–18 years undergoing chemotherapy with available CT/PET-CT scans were included. Anthropometric data (weight, height, arm circumference, triceps skinfold thickness) and HGS were collected. At the third lumbar vertebra (L3), skeletal muscle mass (SMM), skeletal muscle index (SMI), total adipose tissue, and muscle radiodensity were measured; psoas area was assessed at L4. Associations were analyzed using χ2 tests and logistic regression adjusted for sex, age, tumor stage, and type. Forty-eight patients were included (mean age 12.6 ± 3.3 years; 64.6% male); 64.6% had hematologic malignancies and 53.6% had advanced disease. Most were classified as having normal nutritional status (50–64.6%). Hematologic (100%) and gastrointestinal (85.4%) toxicities were most frequent. Low SMM and SMI were correlated with a higher risk of infection (OR 4.20; 95% CI 1.23–14.27 and OR 3.92; 95% CI 1.17–13.20, respectively) and fever (OR 4.05; 95% CI 1.21–13.54 and OR 5.82; 95% CI 1.67–20.25, respectively). After adjustment, only low SMI remained independently associated with fever (OR 4.74; 95% CI 1.09–20.61; *p* = 0.038).

*Conclusions*: Low muscle mass was associated with early infection and fever-related toxicity, suggesting a role of muscle quantity in immune function during pediatric cancer treatment.

**Table:** 

**What is Known:** • *Body composition in children and adolescents changes during chemotherapy is associated with treatment-related toxicity*. • *Muscle status is crucial for chemotherapy tolerance and treatment response*.		
**What is New:** • *Low skeletal muscle mass was associated with a higher risk of toxicities, including fever*. • *No association was found between anthropometric parameters, HGS and toxicities, indicating that further studies are needed*.

**Supplementary Information:**

The online version contains supplementary material available at 10.1007/s00431-026-06882-x.

## Introduction

Pediatric cancer, affecting individuals between 0 and 19 years of age, includes a group of diseases marked by the uncontrolled growth of abnormal cells. In this age group, embryonal-type cancers predominate, typically involving cells of the hematopoietic system and supporting tissues. The most common cancer types in this population is leukemias, central nervous system tumors, and lymphomas [1]. It is estimated that by 2030, approximately two billion new pediatric cancer cases will be diagnosed worldwide among individuals aged 0 to 19 years. Of these, about 51.3% are expected to occur in boys and 48.7% in girls, indicating a higher prevalence in males and highlighting the magnitude of the disease in the pediatric population [2].

Childhood and adolescent cancer negatively affect nutritional status, impairing muscle quantity and quality, with loss of mass and strength. Contributing factors include treatment effects, inactivity, and inadequate intake [3–6]. Nutritional status influences treatment outcomes [7–9], and muscle status is particularly important for chemotherapy tolerance and response [10, 11]. Severe skeletal muscle depletion is associated with worse outcomes, including increased infections and higher chemotherapy toxicity [12–16], highlighting the need for appropriate assessment of nutritional status and body composition.

Handgrip strength (HGS) is recognized as an indicator of nutritional status and a low-cost method used to assess muscle function [17, 18]. It is a simple and reliable measure that has been used as a marker of overall health status and quality of life in various clinical populations and can reflect total body strength in healthy children and adolescents [19, 20]. A recent study observed a strong correlation between HGS and mid-arm muscle circumference (MAMC), indicating that HGS may also reflect muscle mass [21].

Computed Tomography (CT) is considered the gold standard imaging technique for body composition analysis [22]. In oncology, CT scans are routinely performed for diagnosis and follow-up, representing an opportunity to assess patients’ body composition [23]. Its high-resolution, three-dimensional imaging allows for evaluation of both the quantity and quality of skeletal muscle, through measures of muscle tissue density [4, 22, 24]. However, the use of CT for body composition assessment is still relatively new in pediatrics, representing a distinguishing feature in this context.

Studies in pediatric oncology have shown that CT-derived body composition parameters are associated with treatment tolerance. Baseline and longitudinal assessments of skeletal muscle mass and adipose tissue in children with lymphoma, rhabdomyosarcoma, and bone and soft tissue sarcomas have demonstrated associations with higher chemotherapy-related toxicity and poorer survival, highlighting the clinical relevance of skeletal muscle and its changes over the course of treatment. However, these studies did not evaluate chemotherapy-related infections, such as febrile neutropenia or sepsis, as primary outcomes, nor did they assess hematologic toxicities using standardized grading systems [25–27]. In contrast, anthropometric indicators of nutritional status have been consistently associated with a higher risk of febrile neutropenia, infections, and hematologic toxicity in children and adolescents with cancer, without integration of these findings with CT-derived body composition assessments [28–30]. Thus, a methodological and clinical gap remains regarding the combined role of CT-derived body composition, muscle strength, and nutritional status in predicting treatment-related infections and toxicities in pediatric oncology.

In this context, the muscle quality index (MQI), defined as the ratio between muscle strength and muscle mass, has been used to assess muscle functional efficiency and has been significantly associated with adverse outcomes [31–33]. Furthermore, Pereira et al. [6] proposed the strength-to-muscle radiodensity index (SMRi), defined as the ratio between handgrip strength (HGS) and skeletal muscle density (SMD), as a potential marker of muscle quality and demonstrated its prognostic value in predicting mortality in adult patients with colorectal cancer. These findings highlight the importance of an integrated assessment of muscle quality that considers not only muscle strength but also structural muscle characteristics, as changes in muscle composition or attenuation may occur independently of measurable changes in muscle mass, thereby affecting force-generating capacity [6].

Accordingly, the present study aimed to evaluated whether CT-derived body composition parameters and nutritional status are independent predictors of chemotherapy-related infections and hematologic toxicity during the early phases of treatment in pediatric cancer patients.

## Methods

### Study design and population

This was a prospective observational cohort study conducted with children and adolescents diagnosed with malignant neoplasms and undergoing treatment at the National Cancer Institute (INCA). All eligible patients were consecutively enrolled between September 2021 and August 2024.

Children aged 7 to 18 years were eligible for inclusion. Participants were included if they met all the following criteria:

Inclusion criteria

- Confirmed diagnosis of cancer;
- Undergoing chemotherapy;
- Having undergone, within 60 days of the data collection date, abdominal or pelvic computed tomography (CT) or positron emission tomography (PET) with images available at the L3 vertebral level;

Exclusion criteria

- Clinical conditions limiting handgrip strength (HGS) assessment;
- Receiving palliative care;
- Disease control status;
- Congenital syndromes;
- Limb amputations;
- Admission to an intensive care unit (ICU);
- Undergoing chemotherapy protocols without curative intent (second-line, third-line, or salvage regimens);

### Ethical aspects

The study was approved by the INCA Research Ethics Committee (CEP-INCA; No. 5.009.057/2021), with two amendments (No. 5.427.893/2022 and No. 5.837.967/2022). Authorization from parents or guardians for the participation of minors was obtained, as well as signatures of three copies of the Informed Consent Form (ICF) and three copies of the Assent Form (AF), in accordance with ethical research guidelines. The procedures performed in this study adhere to the principles of the Declaration of Helsinki.

### Criteria established for classification of oncologic disease risk

Due to the wide variety of neoplasms present in the study population, an oncologic risk classification system was developed to establish a uniform criterion for assessing disease status. Participants were categorized as having advanced disease (lymphomas stage III or IV; high-risk leukemia/lymphoma; and metastatic solid tumors) or non-advanced disease (lymphomas stage I or II; low- or intermediate-risk leukemia/lymphoma; and non-metastatic solid tumors).

### Anthropometric assessment and nutritional status classification

Anthropometric data were collected by a single trained evaluator during one assessment, within up to 60 days of the CT scan. During the evaluations, the presence of clinical signs of edema or other conditions capable of altering body measurements was checked, and any occurrences were recorded. Additionally, medical records were reviewed for information regarding hyperhydration protocols or ongoing hydroelectrolytic alterations to control potential influences on anthropometric parameters.

Children and adolescents underwent anthropometric assessment including body weight, height and handgrip strength (HGS). Based on these measurements, body mass index-for-age (BMI/A), height-for-age (H/A). Body weight and height were measured using a calibrated digital scale (precision: 0.10 kg) and a stadiometer (precision: 0.10 cm), with participants wearing light clothing and no shoes. BMI was calculated by dividing weight (kg) by height (m^2^).

Anthropometric Z-scores for BMI/A and H/A were calculated using WHO AnthroPlus software (version 3.2.2, January 2011) and classified according to World Health Organization (WHO) growth charts. Undernutrition was operationally defined based on the BMI/A index (z <  − 2). BMI/A was categorized as undernutrition (z <  − 2), nutritional risk (≥ − 2 and <  − 1), normal (≥ − 1 and <  + 1), overweight (≥ + 1 and <  + 2), and obese (≥ + 2). Height-for-age was categorized as severely stunted (z <  − 3), stunted (≥ − 3 and <  − 2), and normal (≥ − 2) [34, 35]. Nutritional status classification was based on BMI/A categories, while height-for-age was analyzed independently and was not incorporated into a composite nutritional classification.

HGS was measured using a Jamar hydraulic hand dynamometer (2.00 kg scale; Sammons Preston™, Canada). Participants were positioned with the shoulder abducted and the elbow flexed at 90°, and were instructed to exert maximal grip strength in response to a verbal command. Three measurements were obtained for each upper limb, and the highest value was used for analysis. Given the absence of validated HGS cut-off points for the pediatric population evaluated and to allow a more accurate interpretation of MQI, was used strength-to-muscle radiodensity index (SMRi), defined as the ratio between handgrip strength (HGS) and skeletal muscle density (SMD). The mean was used to stratify the sample into two groups (values below vs. above the mean), ensuring comparability between groups and enabling the analysis of associations with chemotherapy-related toxicities.

### Body composition assessment by computed tomography

Computed tomography images used in this study were obtained exclusively from clinically indicated examinations performed as part of initial staging and treatment planning, with no additional radiation exposure for research purposes. Skeletal muscle mass (SMM) at the level of the third lumbar vertebra (L3)—including the psoas, erector spinae, quadratus lumborum, transversus abdominis, internal and external obliques, and rectus abdominis—was assessed from CT images. Subcutaneous, visceral, and intramuscular adipose tissues were also measured and summed to calculate total adipose tissue (TAT). Additionally, total psoas muscle area (PMA) at the level of the fourth lumbar vertebra (L4) and skeletal muscle radiodensity (SMD) were evaluated using mean Hounsfield unit (HU) values.

Different vertebral landmarks were selected according to established methods. The L3 level is the reference standard for estimating whole-body skeletal muscle mass due to its strong correlation with total muscle volume [36, 37], whereas psoas muscle area (PMA) is commonly measured at L4, where better anatomical definition improves reproducibility and has been increasingly adopted in pediatric CT-based studies [27, 36–40]. CT image segmentation was performed by a single trained investigator, and a sample was independently reviewed by a second evaluator to ensure accuracy. All images were coded and analyzed prior to toxicity data collection to avoid bias in the toxicity analyses.

Slice-O-Matic software (version 5.0, TOMOVISION, Montreal, Canada) was used to analyze images and calculate tissue areas according to Hounsfield attenuation values: − 29 to + 150 HU for skeletal muscle, − 190 to − 130 HU for subcutaneous and intramuscular fat, and − 150 to − 50 HU for visceral fat [41]. Muscle area was normalized by height (m^2^) and reported as skeletal muscle index (SMI). Due to the absence of validated cut-off points for SMM (cm^2^), SMI (cm^2^/m^2^), TAT (cm^2^) and SMD, in this population, an internal classification criterion was required. As the variables did not exhibit a normal distribution, the median was used to stratify the sample into two groups (values below vs. above the median), ensuring comparability between groups and enabling the analysis of associations with chemotherapy-related toxicities.

### Assessment of chemotherapy-related toxicities

Toxicities were assessed during the first 90 days following the initiation of chemotherapy (early-treatment), corresponding to approximately two chemotherapy cycles. Events were categorized into four groups: gastrointestinal, hematological, infectious, and other toxicities, the latter including isolated events observed during follow-up. Data were extracted from electronic medical records based on documentation from physicians and the nursing, nutrition, and physical therapy teams and recorded using a standardized data collection form. Chart review was performed by one investigator and independently verified by a second researcher to ensure data accuracy.

All toxicities were treated as dichotomous variables (yes/no) according to the Common Terminology Criteria for Adverse Events (CTCAE), version 5.0 [42], except fever [43]. Hematological parameters, including hemoglobin, platelets, neutrophils, leukocytes, and lymphocytes, were obtained from laboratory tests performed during chemotherapy and classified as grade [42]. For the dichotomous classification of the hematological parameters, "yes" was considered when values were below grade 1 (Supplemental tables).

A temperature of 37.7 °C was classified as a fever according to institutional protocol and with the aim of preventing complications, since this population has a high risk of febrile neutropenia [43]. Infectious events included episodes of febrile neutropenia, clinically diagnosed infections (such as central venous catheter–related infection and respiratory infection), according to the treating physicians’ clinical assessment. Event classification was based on clinical and laboratory information recorded in the medical charts, and microbiological confirmation was not required for outcome definition [42].

### Statistical analyses

All children and adolescents included had complete data for the variables analyzed. Therefore, there were no missing data or need for data imputation.

Data normality was assessed using the Shapiro–Wilk test. Continuous variables were presented as mean and standard deviation (SD) when normally distributed, or as median and interquartile range (IQR) when non-normally distributed. Categorical variables were presented as absolute and relative frequencies. Comparisons of anthropometric and body composition variables between groups, stratified by sex and age group, were performed using analysis of variance (ANOVA) or the Kruskal–Wallis test, depending on data distribution. Correlations between body composition variables were evaluated using Spearman’s correlation coefficient. Associations between body composition and toxicity were analyzed by the Chi-square test, and variables with *p* < 0.20 were included in univariate and multivariate logistic regression models, in accordance with the prerequisites for statistical testing, including assessment of multicollinearity, absence of outliers, and the minimum number of cases per dependent variable. Model adequacy was evaluated using a stepwise approach and the Nagelkerke R^2^. Adjustment variables included sex, age, disease stage, and tumor type. The significance level was set at *p* < 0.05 with a 95% confidence interval (95% CI). Analyses were performed using the Statistical Package for the Social Sciences (SPSS), version 22.

Power estimation followed the approach described by Hsieh, Bloch, and Larsen [44] for logistic regression, incorporating the total sample size, outcome prevalence, and the observed adjusted odds ratios. Assuming a two-sided significance level of 5% (α = 0.05) and a conservative exposure prevalence of approximately 50%, these adjusted effect sizes correspond to an estimated statistical power of approximately 80–90% for odds ratios ≥ 4.5, and 70–80% for odds ratios around 4.0.

## Results

### Characteristics of the study population

**Fig. 1:**
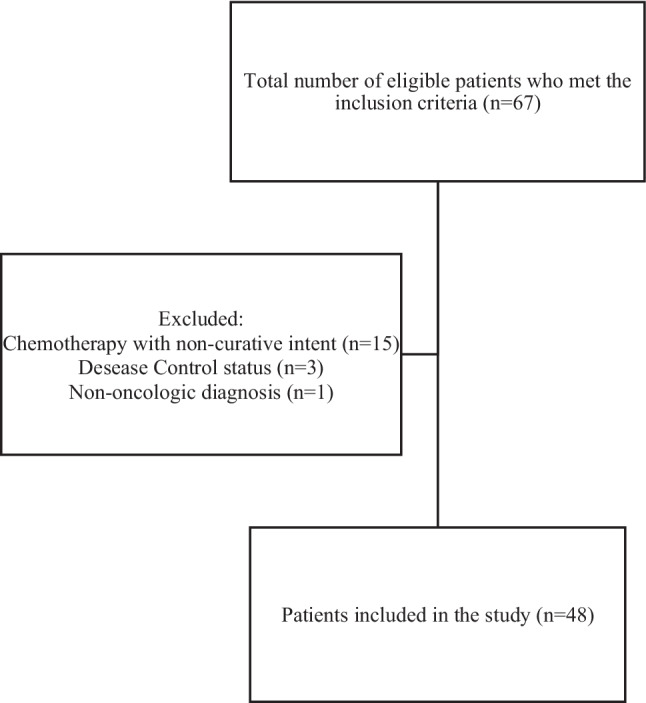
Total number of children and adolescents with cancer undergoing chemotherapy included in the study

Based on the established eligibility criteria, 48 patients were included in this study (Fig. 1). The mean age was 12.56 ± 3.26 years, and males predominated (64.6%; *n* = 31). Hematologic malignancies accounted for 64.6% of cases (*n* = 31), while 35.4% (*n* = 17) had solid tumors. Most patients (56.3%; *n* = 27) presented with advanced-stage disease (Table 1).

**Table 1:** General characteristics and nutritional status classifications of the study population (*n* = 48)

Variables	Overall (%)*N* = 48	7–9 years (%) *N* = 11	10–14 years (%) *N* = 20	15–18 years (%) *N* = 17
Age (years)- Mean (SD)				
	12.6 (3.3)	7.9 (0.8)	12.2 (1.2)	16.1 (1.0)
Sex				
Male	31 (64.6)	8 (72.7)	11 (55)	12 (70.6)
Female	17 (35.4)	3 (27.3)	9 (45)	5 (29.4)
Type of neoplasm				
Hematologic	31 (64.6)	4 (36.4)	13 (65)	14 (82.4)
Solid tumor	17 (35.4)	7 (63.6)	7 (35)	3 (17.6)
Cancer stage*				
Advanced	27 (56.3)	4 (36.4)	13 (65)	10 (58.8)
Not advanced	21 (43.8)	7 (63.6)	7 (35)	7 (41.2)
BMI/Age classification				
Undernutrition	2 (4.2)	1 (9.1)	-	1 (5.9)
Nutritional risk	10 (20.8)	1 (9.1)	6 (30)	3 (17.6)
Normal	25 (52.1)	6 (54.5)	10 (50)	9 (52.9)
Overweight	7 (14.6)	2 (18.2)	2 (15)	2 (11.8)
Obese	4 (8.3)	1 (9.1)	1 (5)	2 (11.8)

*BMI* Body Mass Index

*Cancer stage was classified as “advanced” (lymphomas staged III and IV; high-risk leukemia/lymphoma; and metastatic solid tumors) and “not advanced” (lymphomas staged I and II; low- or intermediate-risk leukemia/lymphoma; and non-metastatic solid tumors)

The most prevalent tumor type was non-Hodgkin lymphoma (25%; *n* = 12), followed by Hodgkin lymphoma (14.6%; *n* = 7), leukemia (12.5%; *n* = 6), combined lymphoma/leukemia (12.5%; *n* = 6), rhabdomyosarcoma (8.3%; *n* = 4), other soft-tissue sarcomas (6.3%; *n* = 3), and other tumor types (22.9%; *n* = 11). The most frequently used chemotherapy protocol was BFM-NHL (41.7%; *n* = 20) (vincristine, ifosfamide, cytarabine, doxorubicin, L-asparaginase, cyclophosphamide, etoposide, and methotrexate), followed by EPSSG (14.6%; *n* = 7) (ifosfamide, doxorubicin, vincristine, actinomycin D, and cyclophosphamide).

Regarding corticosteroid exposure, 33 patients (68.8%) received chemotherapy regimens that included corticosteroids as part of the therapeutic protocol, whereas 15 patients (31.3%) were treated with corticosteroid-free regimens. At the time of anthropometric assessment, 24 patients (50%) were evaluated at baseline, defined as a few days before the initiation of chemotherapy or during the first treatment cycle. Additionally, 11 patients (22.8%) were assessed after one cycle of chemotherapy and 5 patients (10.4%) after two cycles. Only a small proportion of patients (*n* = 6; 12.5%) were evaluated after four or more chemotherapy cycles. The median interval between patient enrollment and the CT scan was 9.5 days (IQR: 3.2–20.5).

### Nutritional status assessed by anthropometry

Regarding nutritional status, most patients (97.9%) had normal H/A, and adequate nutritional status predominated, accounting for 52.1% of the sample. This pattern was consistent across all age groups (Table 1).

Anthropometric measures increased with age, with significant differences observed for height and HGS. Males had higher HGS values, and this was the only parameter significantly different between sexes (*p* = 0.036). BMI-for-age and height-for-age Z-scores were similar between sexes and across age groups. Significant differences in the HGS/SMD ratio across age groups were found only among boys (*p* < 0.001). Data stratified by sex and age are shown in Table 2.

**Table 2:** Values of anthropometric parameters and body composition by CT divided by age group and sex (*n* = 48)

Variables (Male)	Overall Mean or median (SD or IQR) *N* = 31 (100%)	7–9 years Mean or median (SD or IQR) *N* = 8 (25.81%)	10–14 years Mean or median (SD or IQR) *N* = 11 (35.48%)	15–18 years Mean or median (SD or IQR) *N* = 12 (38.71%)	*p*-valor
Weight (Kg)	49.9 (18.1)	30.2 (9.4)	50.2 (13.9)	62.8 (14.1)	< 0.001ᵅᵇ
Height (cm)	156.1 (18.4)	131.9 (8.4)	158.1 (14.3)	170.3 (6.8)	< 0.001ᵅᵇᶜ
Height/Age (z-score)	0.2 (1.1)	0.5 (0.8)	0.5 (1.3)	−0.3 (0.9)	0.120
BMI (Kg/m^2^)	19.8 (4.1)	17.0 (3.4)	19.8 (3.5)	21.6 (4.3)	0.046ᵇ
BMI/Age (z-score)	0.2 (1.5)	0.3 (1.9)	0.4 (1.2)	−0.4 (1.4)	0.751
HGS (Kg)	21.1 (11.8)	9 (3.4)	16.5 (6.5)	33.3 (6.8)	< 0.001ᵅᵇᶜ
SMM (cm^2^)	103.40 (67.60 to 135)	61.90 (55.44 to 74.38)	90.59 (80.65 to 111.90)	136.50 (113.40 to 158.60)	< 0.001ᵅᵇᶜ
PMA (cm^2^)	17.04 (11.17 to 24.66)	10.12.(8.79 to 13.37)	17.04 (11.33 to 21.70)	25.78 (20.64 to 31.00)	< 0.001ᵇᶜ
SMI (cm^2^/m^2^)	38.91 (37.08 to 42.61)	37.47 (33.57 to 38.82)	38.64 (33.68 to 40.21)	45.11 (38.93 to 57.11)	0.007ᵇ
TAT (cm^2^)	49.90 (35.93 to 160.57)	36.95 (25.45 to 131.14)	112.52 (48.91 to 236.99)	68.35 (32.95 to 153)	0.139
SMD (HU)	40.68 (34.07 to 45.11)	40.20 (37.17 to 50.01)	32.84 (30.81 to 45.11)	42.30 (40.33 to 45.76)	0.214
HGS/SMD	0.52 (0.26)	0.22 (0.09)	0.44 (0.12)	0.79 (0.13)	< 0.001ᵅᵇᶜ
Variables (Female)	Overall Mean or median (SD or IQR) *N* = 17 (100%)	7–9 years Mean or median (SD or IQR) *N* = 3 (17.65%)	10–14 years Mean or median (SD or IQR) *N* = 9 (52.94%)	15–18 years Mean or median (SD or IQR) *N* = 5 (29.41%)	*p*-valor
Weight (Kg)	50.53 (22.01)	26.90 (8.71)	50.38 (19.49)	64.97 (21.50)	0.049ᵇ
Height (cm)	152.41 (15.88)	125.50 (13.43)	155.61 (9.06)	162.80 (7.29)	< 0.001ᵅᵇ
Height/Age (z-score)	0.05 (1.31)	−0.74 (1.07)	0.32 (1.50)	0.02 (1.08)	0.505
BMI (Kg/m^2^)	20.87 (6.29)	16.67 (2.05)	20.30 (5.71)	24.43 (7.80)	0.231
BMI/Age (z-score)	0.38 (1.59)	0.27 (0.73)	0.31 (1.69)	0.57 (2.03)	0.956
HGS (Kg)	15.47 (6.25)	9.67 (4.04)	15.33 (5.10)	19.20 (7.29)	0.107
SMM (cm^2^)	81.76 (68.75 to 101.33)	62.55 (56 to 66.17) *	81.76 (74.20 to 105.60)	90.92 (84.90 to 116.38)	0.037ᵇ
PMA (cm^2^)	13.18 (11.17 to 17.92)	10.35 (9.55 to 12.57) *	14.19 (11.40 to 17.92)	17.22 (12.00 to 22.75)	0.120
SMI (cm^2^/m^2^)	36.88 (31.87 to 40.66)	38.18 (34.75 to 45.45) *	36.88 (30.90 to 40.38)	33.39 (30.64 to 46.35)	0.629
TAT (cm^2^)	115.33 (51.64 to 201.21)	63.54 (21.23 to 96.21) *	115.33 (54.73 to 251.17)	157.66 (78.06 to 380.18)	0.143
SMD (HU)	41.75 (32.72 to 44.96)	43.55 (32.83 to 45.13) *	41.75 (33.36 to 44.10)	36.94 (28.95 to 50)	0.967
HGS/SMD	0.41 (0.19)	0.24 (0.10)	0.40 (0.17)	0.53 (0.22)	0.114

*BMI* Body Mass Index, *MUAC* Mid-Upper Arm Circumference, *TSF* Triceps Skinfold Thickness, *MAMC* Mid-Arm Muscle Circumference, *AMA* Arm Muscle Area, *HGS* Handgrip Strength. *SMM* Skeletal Muscle Mass, *PMA* Psoas Muscle Area, *SMI* Skeletal Muscle Index, *SMD* Skeletal Muscle Density, *TAT* Total Adipose Tissue, *HU* Hounsfield Units—tissue radiodensity

ANOVA with Bonferroni post hoc for values expressed as mean. Kruskal–Wallis with pairwise comparison for values expressed as median. ^a^*p* < 0.05 between 7–9 and 10–14 years; ^b^*p* < 0.05 between 7–9 and 15–18 years; ^c^*p* < 0.05 between 10–14 and 15–18 years

*Due to the small number of patients in the groups, data are presented as minimum and maximum

### Body composition assessed by computed tomography

The analysis of CT-based body composition showed an age-related increase in muscle area, more evident among males (SMM and PMA). In females, SMM was the only parameter that differed across age groups, with higher values in the 15–18-year group compared with the 7–9-year group (*p* = 0.036) (Table 2). Comparison of CT-based body composition showed higher mean/median muscle areas in males, with significant differences for SMM (*p* = 0.041) and PMA (*p* = 0.033).

### Associations between anthropometric variables, body composition, and treatment-related toxicities

Hematologic toxicities were observed in all patients, with lymphopenia being the most common (89.6%), followed by neutropenia (87.5%) and anemia (87.5%). Red blood cell and platelet transfusions were required in 50% of cases due to anemia and thrombocytopenia, respectively. Patients with hematologic malignancies had lower leukocyte (420/mm^3^; IQR: 80–1280 vs. 2520; IQR: 275–4140; *p* = 0.014) and neutrophil counts (32/mm^3^; IQR: 0.4–445 vs. 1194; IQR: 55.5–2581; *p* = 0.005) compared to those with solid tumors. Gastrointestinal toxicities were reported in 85.4% of patients, with nausea, vomiting, oral mucositis, and constipation being the most frequent (Table 3).

**Table 3:** Frequency of toxicities observed during treatment

Toxicities	Frequency N (%)
Hematologic toxicity	48 (100%)
Lymphopenia	43 (89.6%)
Anemia	42 (87.5%)
Neutropenia	42 (87.5%)
Leukopenia	35 (72.9%)
Thrombocytopenia	30 (62.5%)
Gastrointestinal	41 (85.4%)
Nausea	37 (77.1%)
Vomiting	29 (60.4%)
Oral mucositis	19 (39.6%)
Constipation	18 (37.5%)
Diarrhea	13 (27.1%)
Odynophagia	12 (25%)
Anorexia	6 (12.5%)
Dry mouth	6 (12.5%)
Abdominal distension	5 (10.4%)
Dysgeusia	5 (10.4%)
Abdominal discomfort	4 (8.3%)
Typhlitis	2 (4.2%)
Colitis	2 (4.2%)
Anal mucositis	2 (4.2%)
Melena	1 (2.1%)
Pancreatitis	1 (2.1%)
Infection	28 (58.3%)
Fever	26 (54.2%)
Febrile neutropenia	19 (39.6%)
Respiratory infection	11 (22.9%)
Catheter-related infection	9 (18.8%)
Other	15 (31.3%)

Other: itching, fatigue, rash, petechiae, purpura, paresthesia

Patients with lymphopenia had a lower mean BMI/A compared to those without this toxicity (19.69 ± 4.39 vs. 24.33 ± 7.90 kg/m^2^; *p* = 0.046). However, no association was observed between the mean BMI-for-age Z-score and chemotherapy-related toxicities; therefore, these variables were not included in the logistic regression analyses.

Muscle mass, assessed by SMM and SMI, was associated with infection and fever-related toxicities (Table 4). Values below the median were linked to higher risk of infection (SMM: *p* = 0.019; SMI: *p* = 0.024) and fever (SMM: *p* = 0.020; SMI: *p* = 0.004). Since all patients had hematologic toxicities, individual components were analyzed separately. Low SMD was associated with leukopenia (*p* = 0.024), observed in 60% of patients with SMD below the median. No associations were found with other hematologic components or toxicity grades. Although SMI was positively correlated with HGS (rho = 0.493; *p* < 0.001), HGS and HGS/SMD were not associated with toxicities. No significant correlations were found between anthropometric variables, PMA, TAT, and toxicities, so regression analyses were not performed for these parameters.

**Table 4:** Association between infection-related toxicities during chemotherapy and muscle parameters of body composition

Toxicities	SMM (cm^2^)		*p*-valor	SMI (cm^2^/m^2^)		*p*-valor	SMD (HU)		*p*-valor
	> Median	< Median		> Median	< Median		> Median	< Median	
Infection			**0.019**			**0.024**			0.242
Yes	35.7% (10)	64.3% (18)		32.1% (9)	67.9% (19)		42.9% (12)	57.1% (16)	
No	70% (14)	30% (6)		65% (13)	35% (7)		60% (12)	40% (8)	
Fever			**0.020**			**0.004**			0.562
Yes	34.6% (9)	65.4% (17)		26.9% (7)	73.1% (19)		46.2% (12)	53.8% (14)	
No	68.2% (15)	31.8% (7)		68.2% (15)	31.8% (7)		54.5% (12)	45.5% (10)	
Febrile neutropenia			0.376			0.312			0.376
Yes	42.1% (8)	57.9% (11)		36.8% (7)	63.2% (12)		42.1% (8)	57.9% (11)	
No	55.2% (16)	44.8% (13)		51.7% (15)	48.3% (14)		55.2% (16)	44.8% (13)	
Respiratory Infection			0.500			0.625			0.247^b^
Yes	54.5% (6)	45.5% (5)		45.5% (5)	54.5% (6)		36.4% (4)	63.6% (7)	
No	48.6% (18)	51.4% (19)		45.9% (17)	54.1% (20)		54.1% (20)	45.9% (17)	
Catheter-Related Infection			0.500ᵇ			0.324ᵇ			0.500ᵇ
Yes	44.4% (4)	55.6% (5)		33.3% (3)	66.7% (6)		55.6% (5)	44.4% (4)	
No	51.3% (20)	48.7% (19)		48.7% (19)	51.3% (20)		48.7% (19)	51.3% (20)	
Lymphopenia			0.174ᵇ			0.419ᵇ			0.174ᵇ
Yes	46.5% (20)	53.5% (23)		44.2% (19)	55.8% (24)		46.5% (20)	53.5% (23)	
No	80% (4)	20% (1)		60% (3)	40% (2)		80% (4)	20% (1)	
Anemia			0.094ᵇ			0.620ᵇ			0.667ᵇ
Yes	45.2% (19)	54.8% (23)		40.4% (17)	59.5% (25)		50% (21)	50% (21)	
No	83.3% (5)	16.7% (1)		83.3% (5)	16.7% (1)		50% (3)	50% (3)	
Neutropenia			0.667b			0.418ᵇ			0.094ᵇ
Yes	50% (21)	50% (21)		47.6% (20)	52.4% (22)		45.2% (19)	54.8% (23)	
No	50% (3)	50% (3)		33.3% (2)	66.7% (4)		83.3% (5)	16.7% (1)	
Leukopenia			0.259			0.978			**0.024**^**b**^
Yes	45.7% (16)	54.3% (19)		45.7% (16)	54.3% (19)		40% (14)	60% (21)	
No	61.5% (8)	38.5% (5)		46.2% (6)	53.8% (7)		76.9% (10)	23.1% (3)	
Thrombocytopenia			0.233			0.881			0.551
Yes	43.3% (13)	56.7% (17)		46.7% (14)	53.3% (16)		46.7% (14)	53.3% (16)	
No	61.1% (11)	38.9% (7)		44.4% (8)	55.6% (10)		55.6% (10)	44.4% (8)	

*SMI* Skeletal Muscle Index, *SMM* Skeletal Muscle Mass, *SMD* Skeletal Muscle Density

Chi-square and ^b^Fisher’s Exact Test

For CT-assessed body composition, muscle mass below the median (SMM and SMI) was correlated with the risk of infection and fever in univariate analyses. After adjustment for confounders, only low SMI remained associated with fever. Low SMD was associated with higher risk of leukopenia, and this association remained significant after adjusting for sex, age, and cancer stage (Table 5). However, adjustment for tumor type (solid vs. hematologic) rendered the association with leukopenia non-significant (OR = 3.54, CI: 0.69–18.34; *p* = 0.131).

**Table 5:** Logistic regression analysis between body composition variables and toxicities

Variables	Univariate OR (95% IC)	*p*-valor	Multivariate OR (95% IC)	*p*-valor
Low SMM				
Infection	4.20 (1.23–14.27)	**0.022**	2.10 (0.30–14.74)	0.457
Fever	4.05 (1.21–13.54)	**0.023**	2.21 (0.32–15.46)	0.423
Low SMI				
Infection	3.92 (1.17–13.20)	**0.027**	2.44 (0.58–10.32)	0.226
Fever	5.82 (1.67–20.25)	**0.006**	4.74 (1.08–20.68)	**0.038**
Low SMD				
Leukopenia	5.00 (1.17–21.46)	**0.030**	5.50 (1.17–26.00)	**0.031**

*OR* Odds Ratio, *CI* Confidence Interval, *SMI* Skeletal Muscle Index, *SMM* Skeletal Muscle Mass, *SMD* Skeletal Muscle Density

Univariate and multivariate logistic regression. Multivariate analysis: adjusted for sex, age (years), and cancer staging. Statistical significance was considered at (*p* < 0.05)

## Discussion

In the present study, which is observational in nature, the findings reflect associations rather than causal relationships. The assessment of body composition using CT in children and adolescents with cancer revealed that lower muscle mass, defined as SMM/SMI below the median, was associated with an increased risk of infection and fever-related chemotherapy toxicities in early-treatment period. Notably, low SMI was independently associated with a higher risk of fever, regardless of age, sex, or cancer stage. Additionally, low SMD was linked to an increased risk of leukopenia, independent of the confounding factors analyzed.

Despite the predominance of advanced disease, most patients were classified as eutrophic across sexes and age groups. Similar results have been described in other cohorts, with a prevalence of normal BMI ranging from 53 to 55%, even among intermediate- or high-risk patients [26, 45, 46]. These findings indicate that BMI-defined nutritional classification often remains within the normal range, even in more advanced stages. Among the anthropometric variables analyzed, only BMI was significantly associated with lymphopenia. However, BMI has not consistently demonstrated reliability as a predictor of chemotherapy-related toxicity in pediatric populations [26, 46]. For example, Wadhwa et al. [26] reported no association between BMI categories and toxicity occurrence (OR: 1.01; 95% CI: 0.45–2.26; *p* = 0.9).

Chemotherapy protocols containing corticosteroids can alter body weight and composition, influencing anthropometric interpretation. Corticosteroid exposure has been associated with increased adipose tissue and changes in body compartments. Tostes et al. [47] observed significant increases in adipose tissue and intramuscular fat in patients with Hodgkin lymphoma treated with the EURONET protocol. In contrast, no effect on body weight or BMI was found in the present study, likely because assessments occurred before or early in chemotherapy, when corticosteroid exposure was minimal.

Muscle weakness is an important predictor of functional decline, making HGS a relevant tool during cancer treatment [18]. In the present analysis, HGS differed by sex, with higher values observed in boys, and increased progressively with age, as expected during growth and development [21, 48]. In addition to reflecting muscle function, HGS is also associated with qualitative aspects of muscle. In this context, the higher HGS/SMD ratio observed in boys may indicate better muscle quality, possibly related to maturation. Although this ratio has shown prognostic value in adults with cancer [6], evidence in pediatric populations remains limited, highlighting an important gap in the literature.

Evidence indicates that HGS is sensitive to treatment-induced changes [39, 49]. In the present study, although it was not associated with chemotherapy-related toxicities, its correlation with SMI supports its use as a functional surrogate of muscle mass in pediatric patients. In clinical practice, although SMI provides a more direct assessment, its use depends on CT imaging and trained evaluators, which may limit its routine application, making HGS a more feasible alternative. Similarly, in older adults with gastrointestinal cancer, low pre-treatment HGS was not associated with dose-limiting toxicity, with significance observed only for hand–foot syndrome [50]. These findings suggest that muscle strength alone may have limited sensitivity for predicting chemotherapy-related complications, highlighting the need for prospective longitudinal studies in pediatric oncology.

In this study, low SMM and SMI were associated with increased susceptibility to immune-related complications, such as infections and fever. Similarly, muscle loss during induction chemotherapy has been associated with a higher incidence of severe adverse events, including invasive fungal infections and grade ≥ 3 toxicities in children and adolescents with acute lymphoblastic leukemia [39]. Additionally, in children and young adults with cancer, a negative correlation between days of neutropenia and PMA suggests a relationship between muscle mass and hematopoietic recovery [16]. Taken together, these findings indicate that lower muscle mass is associated with alterations in immune function and increased hematologic toxicity, although without evidence of a direct causal relationship.

SMD was associated with an increased risk of leukopenia, even after adjustment for clinical variables. Similarly, Wadhwa et al. [26] reported that higher SMD at diagnosis was linked to a lower risk of severe hematologic toxicities, independent of BMI and body surface area (BSA), although no association was observed with SMI. In our analysis, however, the association between low SMD and leukopenia lost significance after adjustment for tumor type, likely reflecting the higher frequency and severity of cytopenias in hematologic malignancies compared with solid tumors [50, 51].

Reductions in muscle mass and quality may impair immune function, increasing susceptibility to infections and cytopenias. Skeletal muscle plays an active role in immune regulation through modulation of inflammatory pathways in a bidirectional relationship: chronic inflammation promotes muscle atrophy, whereas preserved muscle supports immune competence. Disruption of this balance may impair myokine signaling, favor a pro-inflammatory state, and reduce immune cell regeneration, reinforcing the importance of muscle health for immune function [52].

Given the predominance of hematologic malignancies in the sample, it is possible that the initial association between low SMD and leukopenia was driven primarily by tumor-related factors rather than muscle quality alone. Therefore, this finding should be interpreted with caution, particularly considering the limited sample size. Future longitudinal studies with larger and more balanced cohorts by tumor type, as well as stratification by the severity of hematologic toxicity, are needed to clarify the independent role of muscle radiodensity in the severity or persistence of leukopenia.

In children and adolescents with cancer, BSA showed weak correlations with CT-derived parameters (SMI, SMD. TAT), indicating it does not adequately reflect body composition [26]. In adults with small cell lung cancer, chemotherapy doses adjusted for SMI were associated with increased risk of severe hematologic toxicity [53]. These findings highlight BSA limitations for dose adjustment and support SMI as a promising alternative for more individualized dosing, although further validation and imaging availability are required. Additionally, low SMI may help identify patients at higher risk of infection, contributing to monitoring strategies and early risk stratification at treatment initiation.

Few studies have evaluated the relationship between nutritional assessment, muscle strength, CT-based body composition, and chemotherapy toxicity in pediatric populations, resulting in limited comparative data and highlighting the novelty of the present study [26, 27, 37, 38, 47]. This study includes a heterogeneous cohort encompassing both solid and hematologic tumors, providing broader insight across different cancer types.

The limitations of this study include the lack of validated anatomical landmarks for CT-based body composition analysis in the pediatric population, the absence of established reference values, the small sample size, and the lack of dietary intake assessment. The small sample size and median-based categorization may limit the generalizability of the findings, reduce statistical power and obscure potential dose–response relationships. In addition, reliance on toxicity data extracted from medical records restricted more comprehensive analyses, and inclusion based on the availability of CT/PET images may introduce selection bias. The heterogeneity of tumor types and chemotherapy protocols may have introduced residual confounding, potentially limiting the identification of tumor-specific associations. About 25% of the population had an interval between 21–60 days between the CT exam and the nutritional assessment represents a limitation, as pediatric oncology patients may experience rapid changes in body composition and nutritional status during the course of the disease and treatment, and variations may have occurred during this period. Finally, body composition was assessed at a single time point, which does not capture dynamic changes in muscle mass and quality during treatment.

## Conclusion

This study demonstrated that low SMI was associated with a higher risk of chemotherapy-related toxicities, particularly infection- and fever-related events, suggesting a potential role of muscle tissue in immune function. No associations were observed with anthropometric parameters or handgrip strength. These findings reinforce the potential clinical value of body composition assessment at the beginning of treatment for risk stratification. However, given the exploratory nature and single-center design, the results should be interpreted with caution and require validation in larger, multicenter studies to confirm these associations and establish more robust clinical recommendations.

## Supplementary Information

Below is the link to the electronic supplementary material.

Supplementary file1 (DOCX 20 KB)

## Data Availability

No datasets were generated or analysed during the current study.

## Abbreviations

AMA: Arm Muscle Area
BFM LNH: Chemotherapy protocols
BMI/A: Body Mass Index by Age
BSA: Body Surface Area
Chemo: Chemotherapy
CT: Computed Tomography
EPSSG: Chemotherapy protocols
H/A: Height for Age
HGS: Handgrip strength
HL: Hodgkin’s Lymphoma
HU: Hounsfield unit
MAMC: Mid-Arm Muscle Circumference
MQI: Muscle Quality Index
MUAC: Mid-Upper Arm Circumference
PMA: Psoas Muscle Area
PMI: Psoas Muscle Index
SD: Standard Deviation
SMD: Skeletal Muscle Density
SMI: Skeletal Muscle Index
SMM: Skeletal Muscle Mass
SMRI: Strength to Muscle Radiodensity Index
TAT: Total Adipose Tissue
TSF: Triceps Skinfold Thickness
