# Supplementary material for: CT-Based body composition and nutritional status as predictors of early-treatment chemotherapy-related infections and hematologic toxicity in pediatric cancer: a prospective study
Source: Eur J Pediatr. 2026 Mar 25;185(4):216. doi: 10.1007/s00431-026-06882-x (PMC13018010; doi:10.1007/s00431-026-06882-x)
Supplement: Supplementary file 1 — Supplementary file1 (DOCX 20 KB) [file 431_2026_6882_MOESM1_ESM.docx]

| Toxicity | Grade I* | Grade II | Grade III | Grade IV | Grade V |
| --- | --- | --- | --- | --- | --- |
| Anemia | Hb < 10 g/dl | Hb <10-8,0 g/dl | Hb <8,0 g/dl | Life-threatening consequences; urgent intervention indicated | Death |
| Lymphopenia | <LLN - 800/mm³; | <800 - 500/ mm³; | <500 - 200/ mm³; | <200/ mm³; | - |
| Neutropenia | <LLN - 1500/mm³; | <1500 - 1000/ mm³; | <1000 - 500/ mm³; | <500/ mm³; | - |
| Leukopenia | <LLN - 3000/mm³; | <3000 - 2000/ mm³; | <2000 - 1000/ mm³; | <1000/ mm³; | - |
| Thrombocytopenia | <LLN - 75,000/mm³; | <75,000 - 50,000/ mm³; | <50,000 - 25,000/ mm³; | <25,000/ mm³; | - |

Table 1: Hematological toxicities according to CTCAE v5.0 and their classification by grade

Hb: hemoglobin; LLN: Lower Limit of Normal. * For the dichotomous classification, "yes" was considered when values ​​were below grade 1.

Table 2: Definition of toxicities and their classification as dichotomous variables (yes/no):

| Toxicity | Definition | Reference |
| --- | --- | --- |
| Respiratory infection | A disorder characterized by an infectious process involving the upper respiratory tract (nose, paranasal sinuses, pharynx, larynx, or trachea). Moderate symptoms and oral intervention indicated. | 42 |
| Constipation | A disorder characterized by irregular and infrequent or difficult evacuation of the bowels. Occasional or intermittent symptoms and use of dietary modification. | 42 |
| Diarrhea | A disorder characterized by an increase in frequency and/or loose or watery bowel movements. Increase of <4 stools per day over baseline. | 42 |
| Mucositis oral | A disorder characterized by ulceration or inflammation of the oral mucosal. Mild symptoms; intervention not indicated. | 42 |
| Anal mucositis | A disorder characterized by ulceration or inflammation of the mucous membrane of the anus. Mild symptoms; intervention not indicated. | 42 |
| Colitis | A disorder characterized by inflammation of the colon. Clinical or diagnostic observations only; intervention not indicated. | 42 |
| Nausea | A disorder characterized by a queasy sensation and/or the urge to vomit. Loss of appetite without alteration in eating habits. | 42 |
| Vomiting | A disorder characterized by the reflexive act of ejecting the contents of the stomach through the mouth. Mild symptoms; intervention not indicated | 42 |
| Anorexia | Disorder characterized by a loss of appetite. Any reduction in food intake. | 42 |
| Abdominal distension | A disorder characterized by swelling of the abdomen. Clinical or diagnostic observations only; intervention not indicated. | 42 |
| Pancreatitis | A disorder characterized by inflammation of the pancreas with no documented pancreas infection, with enzyme elevation. | 42 |
| Dysgeusia | A disorder characterized by abnormal sensual experience with the taste of foodstuffs; it can be related to a decrease in the sense of smell. Altered taste but no change in diet | 42 |
| Typhlitis | A disorder characterized by necrotizing enterocolitis in neutropenic patients. Symptomatic (abdominal pain, fever, change in bowel habits with ileus) | 42 |
| Abdominal discomfort/ pain | A disorder characterized by a sensation of marked discomfort in the abdominal region. Mild pain. | 42 |
| Fever | A disorder characterized by elevation of the body's temperature above the upper limit of normal – > 37.7º C | 43 |
| Febrile neutropenia | A disorder characterized by an ANC <1000/mm3 and a sustained temperature of >=38º C for more than one hour. | 42 |
| Catheter related infection | A disorder characterized by an infectious process that arises secondary to catheter use.  Localized; local intervention indicated. | 42 |
| Odynophagia/ oral pain | A disorder characterized by a sensation of marked discomfort in the mouth, tongue or lips. Mild oral pain. | 42 |
| Melena/ Upper gastrointestinal hemorrhage | A disorder characterized by bleeding from the upper gastrointestinal tract (oral cavity, pharynx, esophagus, and stomach). Mild upper gastrointestinal hemorrhage.symptoms; intervention not indicated. | 42 |

ANC: Absolute Neutrophil Count.
